# Pulmonary hypertension in pediatrics: from clinical suspicion to management

**DOI:** 10.1007/s00431-025-06099-4

**Published:** 2025-04-09

**Authors:** Julie Wacker, Maurice Beghetti

**Affiliations:** 1 Pediatric Cardiology Unit, Department of Pediatrics, Gynecology, and Obstetrics, Geneva University Hospitals, Rue Willy Donzé 6, 1211 Genève 14, Geneva, Switzerland; 2 Pulmonary Hypertension Program, Geneva University Hospitals, Geneva, Switzerland

**Keywords:** Pulmonary hypertension, Physiopathology, Congenital heart disease, Developmental lung disorder, Risk stratification

## Abstract

Pediatric pulmonary hypertension differs from adult pulmonary hypertension in many ways, including multifactorial etiologies and comorbidities that can impact diagnosis, response to therapy, and outcome. The main etiologies of pediatric PH are idiopathic pulmonary arterial hypertension (PAH), PAH associated with congenital heart disease (PAH-CDH) and developmental lung disorders. Thorough diagnostic evaluation is necessary to properly classify pulmonary hypertension, find a potential treatable cause, and guide therapy. Diagnosis still relies on invasive hemodynamics that require sedation in most children. Management of pediatric pulmonary hypertension is mainly guided by small-scale studies, expert opinion, and extrapolation of adult data considering the paucity of trials in this population. The aim of this review is to provide an up-to-date summary of current knowledge on pediatric pulmonary hypertension, covering diagnosis to management, and to highlight the key takeaways from the pediatric task force of the 7th World Symposium on Pulmonary Hypertension, particularly regarding classification modifications, risk stratification, and management.

**Table:** 

**What is known:** • *Pediatric pulmonary hypertension is a rare condition, with the main etiologies being idiopathic, associated with congenital heart disease and developmental lung disorders.* • *A risk-oriented treatment approach is recommended, with lower-risk mortality as the therapeutic target. Treatment should be escalated if the treatment response is unsatisfactory.* **What is new:** • *Classification of pulmonary arterial hypertension associated with congenital heart disease is expanded beyond the concept of a shunt.* • *Risk stratification is refined through the use of 25 validated risk factors.*

**What is known:**

• *Pediatric pulmonary hypertension is a rare condition, with the main etiologies being idiopathic, associated with congenital heart disease and developmental lung disorders.*

• *A risk-oriented treatment approach is recommended, with lower-risk mortality as the therapeutic target. Treatment should be escalated if the treatment response is unsatisfactory.*

**What is new:**

• *Classification of pulmonary arterial hypertension associated with congenital heart disease is expanded beyond the concept of a shunt.*

• *Risk stratification is refined through the use of 25 validated risk factors.*

## Introduction

Despite tremendous advances in the comprehension of its physiopathology and management, pulmonary hypertension remains an incurable disease. Several challenges arise when dealing with pulmonary hypertension: from clinical suspicion based on non-specific symptoms of a rare disease to management, which is guided by expert opinions and small studies rather than large-scale randomized controlled trials. In between lies the difficulty of establishing a diagnosis that relies on invasive hemodynamics, particularly in very sick or very small children. Identifying the etiology of pulmonary hypertension is crucial, as some could be amenable to treatment. One of the specificities of pediatric pulmonary hypertension is the complexity of multifactorial processes leading to pulmonary hypertension and the burden of comorbidities that renders the population more heterogeneous and require careful and complete baseline evaluation and the collaboration of multidisciplinary teams to tailor management to the individual.

The Pediatric Task Force of the 7th World Symposium on Pulmonary Hypertension recently provided updates on diagnosis, classification, risk-stratification, and treatment of pediatric pulmonary hypertension. They are incorporated in the present review discussing the main causes of pulmonary hypertension in children, including pulmonary arterial hypertension associated with congenital heart disease (PAH-CHD), idiopathic PAH, and developmental lung disorders. The most significant changes made in these recommendations are described below, including the classification of PAH-CHD, parameters of risk-stratification, and the separation in the treatment algorithm based on the presence or absence of cardio-pulmonary condition.

## Definition and classification

The diagnosis of pulmonary hypertension is based on hemodynamic assessment through heart catheterization, with a mean pulmonary artery pressure (mPAP) greater than 20 mmHg at rest [1]. This was modified during the 6th World Symposium on pulmonary hypertension in 2018, lowering the historical cut-off of 25 mmHg, considering: (1) studies assessing the limits of normal mPAP in healthy subjects, and (2) the prognosis of adult patients with a mPAP between 20 and 24 mmHg [2–4]. Whether this new definition is applicable to children remains to be proven, but it has been changed for the sake of homogeneity.

Of note, this definition is valid for children aged 3 months and older due to the normal transition of fetal isosystemic PAP and elevated pulmonary vascular resistance (PVR) to normal values in healthy neonates. Neonatal pulmonary hypertension in the first 3 months of life is not well defined [5].

Pulmonary hypertension is classified into five groups (Table 1) [6]. The categories regroup conditions with similar pathophysiological mechanisms, clinical presentation, and management.

**Table 1:** Clinical classification of pulmonary hypertension

Group 1: PAH
1.1 Idiopathic
1.1.1 Long-term responders to calcium channel blockers
1.2 Heritable
1.3 Associated with drugs and toxins
1.4 Associated with:
1.4.1 Connective tissue disease
1.4.2 HIV infection
1.4.3 Portal hypertension
1.4.4 Congenital heart disease
1.4.5 Schistosomiasis
1.5 PAH with features of venous/capillary (PVOD/PCH) involvement
1.6 Persistent PH of the newborn
Group 2: PH associated with left heart disease
2.1 Heart failure
2.2 Valvular heart disease
2.3 Congenital acquired cardiovascular conditions leading to post-capillary PH
Group 3: PH associated with lung diseases and/or hypoxia
3.1 COPD and/or emphysema
3.2 Interstitial lung disease
3.3 Combined pulmonary fibrosis and emphysema
3.4 Other parenchymal lung diseases
3.5 Nonparenchymal restrictive diseases
3.6 Hypoxia without lung disease (e.g., high altitude)
3.7 Developmental lung disease
Group 4: PH associated with pulmonary artery obstructions
4.1 Chronic thromboembolic PH
4.2 Other pulmonary artery obstructions
Group 5: PH with unclear and/or multifactorial mechanisms
5.1 Haematological disorders
5.2 Systemic disorders
5.3 Metabolic disorders
5.4 Chronic renal failure with or without hemodialysis
5.5 Pulmonary tumor thrombotic microangiopathy
5.6 Fibrosing mediastinitis
5.7 Complex congenital heart disease

*PAH* pulmonary arterial hypertension, *PH* pulmonary hypertension, *PVOD* pulmonary veno-occlusive disease, *PCH* pulmonary capillary hemangiomatosis

Adapted from (5)

Group 1 is PAH, which requires not only a mPAP > 20 mmHg but also an increase in PVR > 2 WU or indexed PVR > 3 WUm^2^ to reflect pulmonary vascular disease and a low pulmonary arterial wedge pressure (PAWP < 15 mmHg) to rule out a post-capillary component. Of note, PVR is indexed for body surface area in small children but should be reported non-indexed in patients with a body surface area > 1.5 m^2^ [5].

About 40% of PAH in children is associated with congenital heart disease (PAH-CHD). A novel subclassification of children with PAH-CHD has been suggested following an assessment of the Tracking Outcomes and Practice in Pediatric Pulmonary Hypertension (TOPP) registry and incorporated in the new set of recommendations on pediatric pulmonary hypertension [5, 7] (Table 2). The first significant change is the relocation of all atrial septal defects (ASD) in the “coincidental” group, considering that ASD does not cause pulmonary vascular disease during childhood. The second major change is the addition of a new category E, which includes patients who have never had a significant shunt and could not be classified in the previous ABCD subgroups, such as patients with timely repair of transposition of the great arteries who develop PAH. Of note, patients with complex CHD and pulmonary vascular disease who do not meet the requirement for PAH classification, such as those with Fontan circulation, belong to group 5 of the classification.

**Table 2:** Classification of congenital heart disease-associated pulmonary arterial hypertension

Group	Condition
A	Eisenmenger syndrome
B	Left-to-right shunt:
B1	Correctable
B2	Not correctable
C	Coincidental defects, including all isolated ASDs in childhood
D	Corrected CHD
E	CHD without initial shunt, including timely repaired transposition of the great arteries

*ASD* atrial septal defect, *CHD* congenital heart disease

Adapted from (6)

Besides PAH-CHD and idiopathic and heritable PAH, other causes of PAH are extremely rare in children. It is however necessary to rule them out, as some may be amenable to treatment, such as congenital porto-systemic shunt (CPSS) causing portopulmonary hypertension [8, 9]. It is believed that the lack of a liver first-pass in CPSS exposes the pulmonary vasculature to harmful factors, triggering a pulmonary vascular disease [10].

The third most common etiology of pediatric pulmonary hypertension is developmental lung disorder, classified in group 3 pulmonary hypertension associated with lung disease. Earlier epidemiological studies estimated that approximately 10–12% of children with pulmonary hypertension had an associated lung disease; however, this proportion is expected to rise with the improved survival rates of extreme premature infants [11, 12]. Bronchopulmonary dysplasia and congenital diaphragmatic hernia are the most frequent disorders of lung development, but rarer conditions like surfactant protein gene abnormalities, alveolar capillary dysplasia, and *TBX4* mutations can be identified. The complete list of developmental lung disorders associated with pulmonary hypertension can be found elsewhere [5].

One of the complexities in managing children with pulmonary hypertension is the multifactoriality of the disease, with frequent overlapping conditions causing pulmonary hypertension. It is through complete diagnostic assessment and involvement of multidisciplinary team that the different mechanisms underlying the origins of pulmonary hypertension can be unveiled.

## Diagnosis

### Signs and symptoms

There are no pathognomonic symptoms of pulmonary hypertension, which are related to right ventricular failure that often develops late in the course of pediatric pulmonary hypertension. It is therefore very important to raise early suspicion of pulmonary hypertension in children presenting with the following symptoms: dyspnea on exertion, fatigue, palpitations, syncope during or after physical exertion, chest pain on exertion, and hoarseness. Suspicion should also be raised in the presence of a family history of pulmonary hypertension or genetic syndromes known to be associated with it, such as Down syndrome.

On physical examination, an accentuated pulmonary component of the second heart sound, a right ventricular heave, and consequences of right ventricular failure such as abdominal distention and hepatomegaly should be sought [13]. Other signs could orientate towards the underlying cause of pulmonary hypertension, such as digital clubbing in liver disease, lung disease, or Eisenmenger syndrome.

Of note, in children with PAH-CHD, the disappearance of heart failure and pulmonary overcirculation symptoms, along with the decrease in the shunt murmur, suggest a significant increase in PVR.

### ECG

Right axis deviation and signs of right ventricular hypertrophy are common but not always present. In children with PAH-CHD, the ECG will vary depending on the underlying lesion and the evolution of right-sided pressures.

### Chest X-ray

Chest X-ray can show enlargement of the right-sided heart chambers and central pulmonary arteries and tapering of the peripheral vessels. Signs related to a developmental lung disorder can also be present.

### Echocardiography

Pulmonary hypertension can be suspected on transthoracic echocardiography, but diagnosis requires heart catheterization. Echocardiography can (1) detect any CHD, (2) rule out a left-sided heart disease, (3) show indirect signs of increased PA pressure and increased PVR (increased eccentricity index, shortened PA acceleration time, notch on the pulmonary valve doppler), (4) estimate PA pressure (right ventricular systolic pressure on the tricuspid regurgitation jet velocity, mPAP and diastolic PAP on the pulmonary regurgitation jet velocity), and (5) inform on right ventricular size and function (enlarged right ventricle, reduced fractional area change and tricuspid annular plane systolic excursion).

### Heart catheterization

Heart catheterization and the sedation or anesthesia required for the procedure in most children are not without risks in patients with pulmonary hypertension, especially in children aged < 1 year, with worse functional class, prematurity, previous respiratory support, and group 3 pulmonary hypertension [14, 15]. However, this risk can be mitigated when the procedure is carried out in pediatric expert centers [14, 16]. A heart catheterization allows to make the diagnosis of pulmonary hypertension, categorize pulmonary hypertension according to the pre and/or post-capillary components, and perform acute vasoreactivity testing in children with idiopathic or heritable pulmonary hypertension using Sitbon criteria (decrease of mPAP of at least 10 mmHg, mPAP less than 40 mmHg with an unchanged or increased cardiac output) [17, 18]. Responders to acute vasoreactivity testing are treated with high-dose calcium channel blockers, and, provided they remain responders, have a better prognosis than the other subgroups of PAH. In children with a congenital heart disease and pulmonary hypertension, acute vasodilatory testing can help determine operability but suggested hemodynamic criteria lack validation [19].

### Etiological work-up

Once the diagnosis of pulmonary hypertension has been made, etiological work-up is crucial to classify pulmonary hypertension and guide management. Pulmonary hypertension in children is complex, and often multifactorial, requiring a careful appraisal of all the parameters causing pulmonary hypertension.

Considering the high frequency of associated lung disorder in pediatric pulmonary hypertension, a comprehensive assessment should be made, with chest CT, polysomnography, and pulmonary function tests when possible. Ventilation/perfusion scintigraphy (V/Q scan) is performed to rule out chronic thromboembolic pulmonary hypertension. Some centers advocate replacing the V/Q scan with dual-energy CT, as it provides information on lung perfusion simultaneously with CT scan acquisition, combining morphological analysis and functional information on lung perfusion during chest CT acquisition [20–22].

Blood tests aiming at ruling out causes of pulmonary hypertension including immunological testing (connective tissue disease), serological testing (human immunodeficiency virus infection), liver enzymes and function (liver disease), and hypercoagulability (chronic thromboembolic pulmonary hypertension) should be performed.

It is paramount to screen for liver disease or CPSS by means of an abdominal ultrasound [10] .

Genetic factors have been shown to contribute to up to 42% of pediatric-onset PAH, with inherited mutations in known PAH risk genes like *BMPR2, TBX4, NOTCH1,* and *SOX 17* but also about 15% of de novo variants in novel genes with functions in vasculogenesis and remodeling [23, 24]. Some mutations are associated with developmental lung disorders. The impact of individual variants on prognosis and treatment response warrants future research.

Six-minute walk distance and cardiopulmonary exercise testing are performed during the diagnosis work-up. If they do not assess the etiology of pulmonary hypertension, they assess the impact of pulmonary hypertension on the physical capacity of the child and may help for prognostication. They can usually be performed starting at the age of 6. Comorbidities, developmental disorders, and lack of collaboration can preclude the adequate realization of these tests.

Cardiac MRI can help assess the ratio of pulmonary blood flow over systemic blood flow in children with PAH-CHD and measure the three-dimensional right ventricular volume, mass, and ejection fraction. It has a strong sensitivity to detect early signs of pulmonary hypertension and can assist with prognostication [25, 26]. Of note, MRI is not universally available, and its widespread use has been limited by the cost, the length of the exam, and the need for sedation or anesthesia in smaller children.

## Risk stratification

Numerous risk stratification tools have emerged in the last decade and validated in adult PAH populations, like the US Registry to Evaluate Early and Long-Term PAH Disease Management (REVEAL) 2.0 and 2.0 lite risk scores, the Comparative, Prospective Registry of Newly Initiated Therapies for pulmonary hypertension (COMPERA) risk score, or the newly introduced four-strata risk assessment tool in the 2022 ESC guidelines [1, 27–29].

There is no validated risk assessment score in pediatrics. The pediatric task force at the 7th World Symposium on pulmonary hypertension identified the main risk factors ever reported to be associated with outcome in pediatric pulmonary hypertension, and suggested cut-off values of lower risk and high risk for every parameter, based on an exhaustive literature search [5]. This proposed risk stratification tool includes clinical, laboratory, echocardiography, MRI, and hemodynamic variables. More data are needed to confirm the cut-off values and define how to optimally combine and weigh those multiple parameters. Genetic variants can affect the outcome of pediatric pulmonary hypertension, but more data are needed before the genetic background can be incorporated into risk stratification.

## Treatment

The primary goal of treatment should always be the reversal of a potential cause of pulmonary hypertension, such as a treatable liver or lung disease.

General measures include immunization, prevention of dehydration, encouragement of regular physical activity, education on contraception and pregnancy risks, and treatment of iron deficiency. Oxygen, anticoagulation, and diuretics should be discussed on a case-by-case basis.

**Fig. 1:**
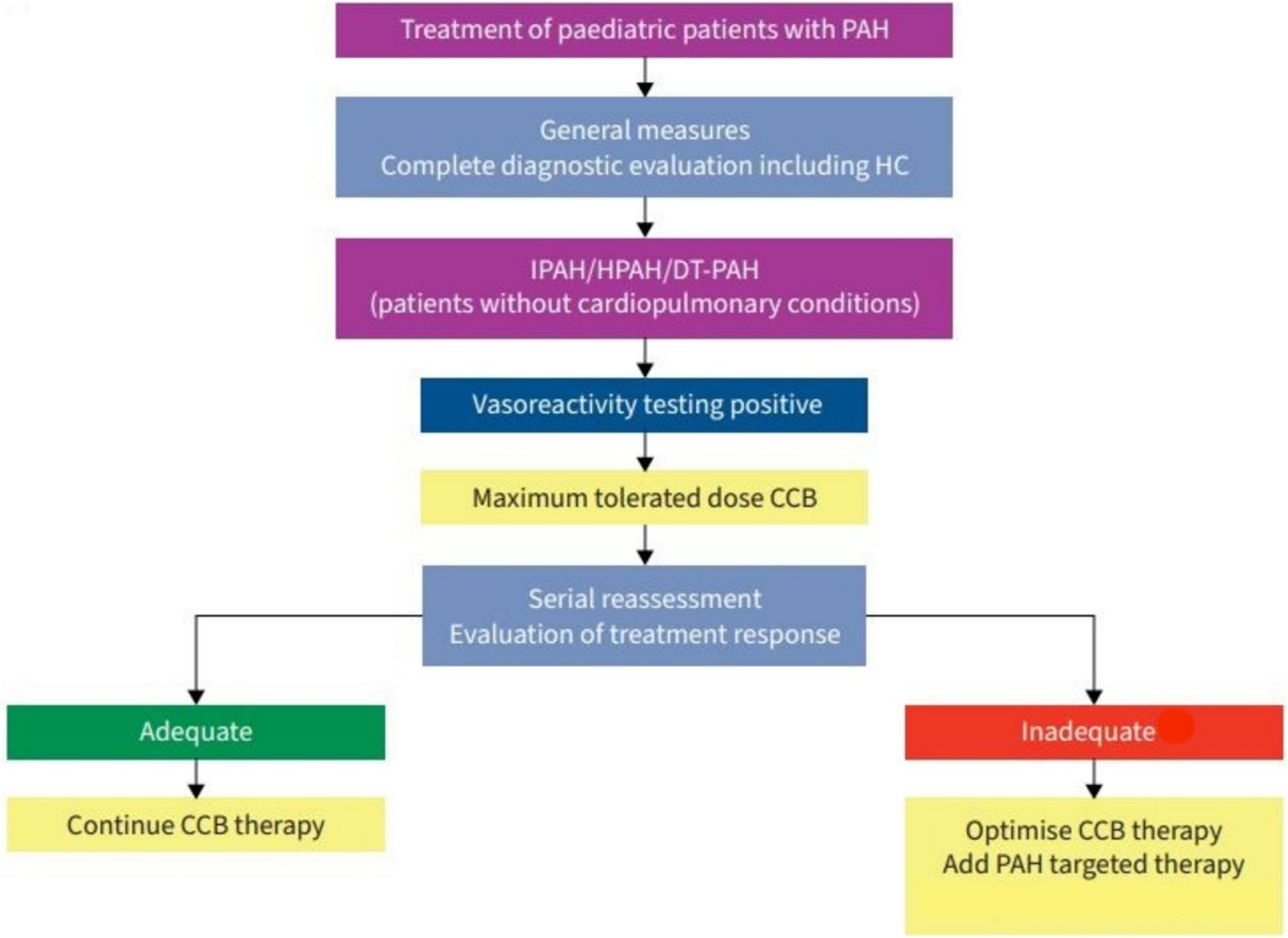
Initial treatment strategy for children with idiopathic, hereditary, and drug-and toxin-induced PAH. CCB, calcium channel blocker; DT-PAH, drug-and toxin-induced pulmonary arterial hypertension; HC, heart catheterization; HPAH, hereditary pulmonary arterial hypertension**;** IPAH, idiopathic pulmonary arterial hypertension; PAH, pulmonary arterial hypertension. Adapted from (6)

**Fig. 2:**
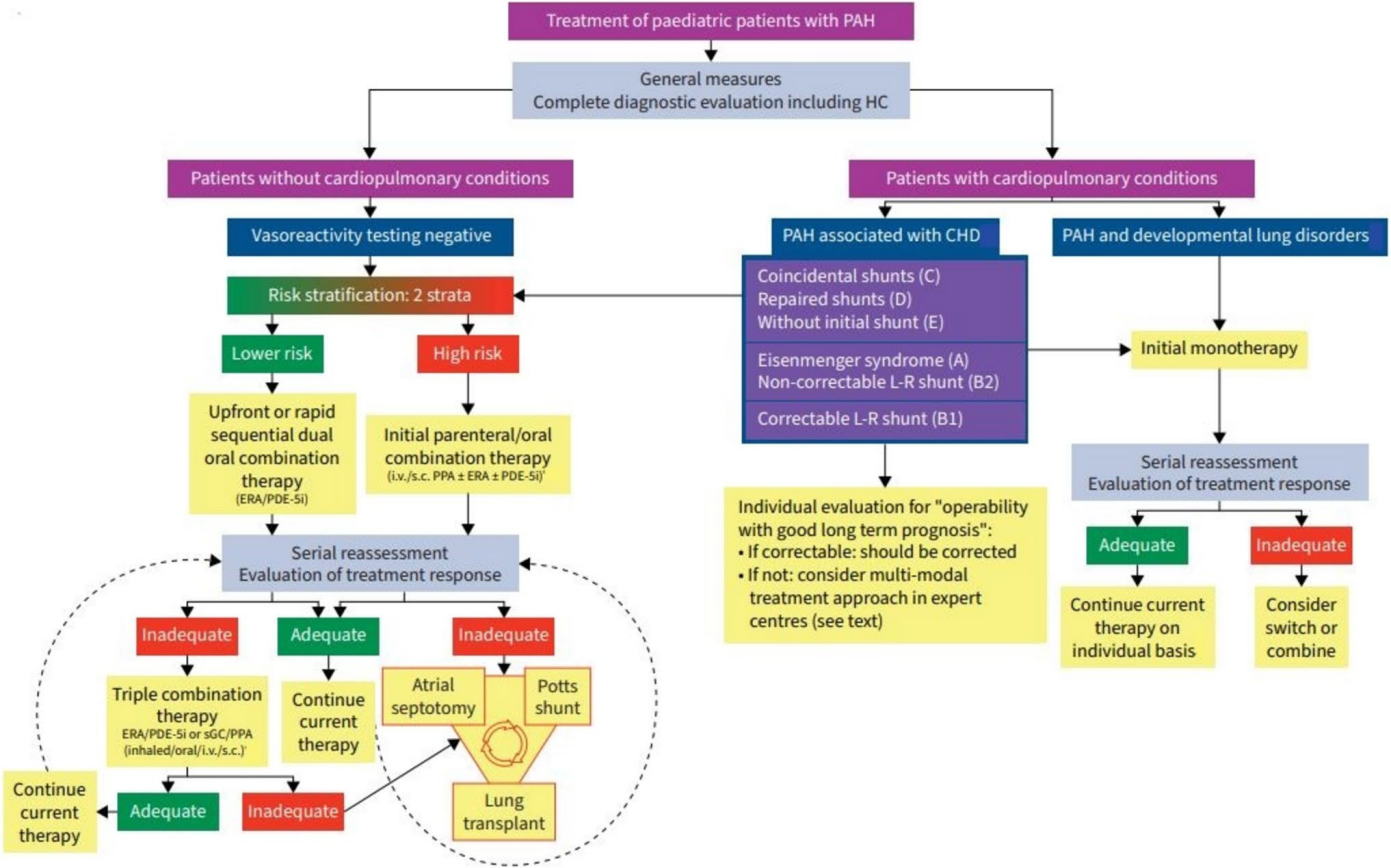
Treatment strategy for children with pulmonary arterial hypertension. CHD, congenital heart disease; ERA, endothelin receptor antagonist; HC, heart catheterisation; I.V., intravenous; L:, left; PAH, pulmonary arterial hypertension; PDE-5i, phosphodiesterase-5 inhibitor; PPA, prostacyclin pathway agent; R, right; S.C., subcutaneous; sGC, soluble guanylate cyclase

The treatment algorithm proposed by the pediatric task force of the 7th World Symposium on pulmonary hypertension is based on real-world data and expert consensus rather than data from multiple randomized controlled trials and is reproduced in Figs. 1 and 2. It is applicable to children with PAH. The collection of robust data on the efficacy of PAH drugs in children has been hampered by the difficulty to perform randomized controlled trials in this population. The rarity of pediatric PAH, its poor outcome, the widespread off-label use of the drugs approved in adults, the lack of validated endpoint, the physiologic changes during childhood, and ethical concerns are just a few of the hurdles to carrying out randomized controlled trials [30].

Pulmonary vasodilators target the three key pathways of pulmonary hypertension identified more than 20 years ago: the endothelin pathway (bosentan, macitentan, ambrisentan), the nitric oxide (NO)/cyclic GMP pathway (sildenafil, tadalafil, riociguat) and the prostacyclin pathway (epoprostenol, treprostinil, selexipag) [31]. A new drug interacting with a fourth pathway, the BMP/TGFb pathway, has recently been approved in adult and will probably be included in the next set of treatment guidelines. Sotatercept, an activin signaling inhibitor, binds activins and growth differentiation factors and restores the balance between pro and antiproliferative BMP pathways [32, 33]. The MOONBEAM clinical trial (NCT05587712) is ongoing in children with PAH aged > 1 year and will inform on the safety and efficacy of sotatercept in children with PAH.

One of the novelties of the 7th World Symposium on pulmonary hypertension pediatric treatment algorithm is the separation in two arms depending on the presence or absence of cardiopulmonary conditions.

### Patients without cardiopulmonary conditions

The first step for patients with idiopathic, hereditary and drug- and toxin-induced PAH is to identify those with a positive acute vasoreactivity testing, using the Sitbon criteria. These responders should be treated with the maximum tolerated dose of calcium channel blockers, with the possible addition of PAH-targeted therapy if there is an unsatisfactory treatment response upon serial reassessment. Indeed, among the 10% of responders at baseline, only about half will remain long-term responders [34].

The non-responders should be treated with upfront combination therapy depending on their risk appraisal: dual oral for patients with a lower risk of death, and initial parenteral/oral combination therapy for the high-risk patients. Treatment goal should be to maintain or reach a lower risk profile. In the case of inadequate treatment response, therapy should be escalated.

For children who have failed available conventional therapies, advanced strategies, including atrial septostomy, reverse Potts shunt and lung transplant, should be considered.

### Patients with cardiopulmonary conditions

In patients with a developmental lung disorder, such as bronchopulmonary dysplasia and congenital diaphragmatic hernia (group 3 pulmonary hypertension), the first aim should be aggressive treatment of the underlying lung disease, and respiratory support optimization. In patients with a severe pre-capillary pulmonary hypertension, which is not easily proven without heart catheterization, there may be a role for a careful introduction of PAH therapies. Some developmental lung disorders, especially bronchopulmonary dysplasia, tend to improve pulmonary hypertension over time, allowing for potential weaning of targeted therapies later on.

Patients with PAH-CHD require a tailored treatment approach depending on the clinical group. Patients with correctable shunt (group B1) should be operated in experienced centers. Those with advanced pulmonary vascular disease, including non-correctable left-to-right shunts and Eisenmenger syndrome (group B2 and group A), should be treated with PAH therapy. No data support upfront combination therapy in this population, but therapy escalation in the case of an unsatisfactory treatment response to initial monotherapy could be considered. PAH patients with small coincidental defects (group C), repaired CHD (group D) and without initial shunt (group E) should be managed as group 1 PAH.

## Conclusion

The complexity, heterogeneity, and multifactorial nature of pulmonary hypertension in children pose diagnostic, classification, and therapeutic challenges. Areas for future research are numerous and are outlined in Table 3. An extensive diagnostic work-up is crucial to understand the mechanisms leading to pulmonary hypertension in a unique individual, in order to develop an adequate treatment plan. Efforts are being made to better define specific pediatric pulmonary hypertension etiologies, such as PAH-CHD and developmental lung disorders.

**Table 3:** Potential future areas of research in pediatric pulmonary hypertension

- Prognosis and treatment of pediatric patients with a baseline mPAP between 20 and 25 mmHg
- Criteria for acute vasoreactivity that can better identify long-term responders to calcium channel blockers
- Definition and treatment of neonatal pulmonary hypertension
- Prognostic relevance of different subgroups of PAH-CHD
- Operability criteria and prognosis for children with PAH-CHD and some degree of pulmonary vascular disease
- Efficacy of targeted therapies in children with a failing Fontan and pulmonary vascular disease
- Efficacy and safety of sotatercept in pediatric PAH, and its place in the treatment algorithm
- Timing and indications for advanced treatment strategies, such as atrial septostomy, Potts shunt, and lung transplant

Risk stratification of pediatric PAH is based on validated parameters, but further studies are needed to confirm the cut-off values and the relative weight of these variables.

The recent advent of sotatercept, a new disease-modifying drug, in the adult PAH therapeutic arsenal generates enthusiasm in the pulmonary hypertension pediatric community, but results of the pediatric trials are awaited. Future studies should also address the indications and timing of advanced strategies, such as atrial septostomy, Potts shunt, and lung transplant in children who fail conventional targeted pulmonary hypertension medical therapies.

Close collaboration between all PAH stakeholders, including patients and their caregivers, pulmonary hypertension physicians, industry, and regulators should help design the future of clinical trials in pediatric pulmonary hypertension and increase evidence-based medicine to guide management in this population.

## Data Availability

No datasets were generated or analysed during the current study.

## Abbreviations

ASD: Atrial septal defect
CPSS: Congenital porto-systemic shunt
mPAP: Mean pulmonary artery pressure
PAH: Pulmonary arterial hypertension
PAH-CHD: Pulmonary hypertension associated with congenital heart disease
PAWP: Pulmonary arterial wedge pressure
PVR: Pulmonary vascular resistance
